# Association of back pain with hypovitaminosis D in postmenopausal women with low bone mass

**DOI:** 10.1186/1471-2474-14-184

**Published:** 2013-06-12

**Authors:** Ariane Viana de Souza e Silva, Paulo Gustavo Sampaio Lacativa, Luis Augusto Tavares Russo, Luiz Henrique de Gregório, Renata Alexandra Calixto Pinheiro, Lizanka Paola Figueiredo Marinheiro

**Affiliations:** 1 Instituto Fernandes Figueira / Fundação Oswaldo Cruz (FIOCRUZ), Rio de Janeiro, Brazil; 2 CCBR Brasil - Center for Clinical and Basic Research, Rua Mena Barreto 33 Botafogo, Rio de Janeiro, Brazil

**Keywords:** Hypovitaminosis D, Back pain, Newwit/Cummings questionnaire

## Abstract

**Background:**

Back pain is a major public health problem due to its high frequency, to the resulting activity constraint, and the need for surgery in many cases. Back pain is more frequent in women than men, mainly in postmenopausal women. High prevalence of hypovitaminosis D has been detected in postmenopausal women, and it is associated with decreased bone mass, sarcopenia, vertebral fractures, and inflammation, which can be related to back pain.

**Methods:**

The relation between back pain and hypovitaminosis D was evaluated in this study, as well the difference regarding the number of bedridden days, number of days away from work, and daily activities limitation between women with and without hypovitaminosis D. This study reviewed baseline data from an interventional phase III multicenter trial in low bone mass postmenopausal women. The study included demographic data, 25OHD determinations, Newitt/Cummings questionnaire on back pain, and vertebral fracture identified thought X-ray evaluation.

**Results:**

The trial included 9354 participants, but only 9305 underwent all the evaluations. The age median was 67 (60 - 85 years old) and age at menopause was 49 (18 - 72 years). Hypovitaminosis D was found in 22.5% of the subjects, 15.3% of them had vertebral fractures, 67.5% with back pain, and 14.8% reduced their daily activities in the previous six months. Subjects with hypovitaminosis D, compared to those without hypovitaminosis D, reported more back pain (69.5 v 66.9%, p: 0.022), more cases of severe back pain (8.5% v 6.8%, p: 0,004), higher limitation in their daily activities (17.2 v 14.0%, p: 0.001), and more fractures (17.4 v 14.6%, p: 0,002); also, they had more trouble to perform daily activities addressed in the Newwit/Cummings questionnaire.

**Conclusion:**

Hypovitaminosis D was related to back pain, to its severity, and to difficulty in perform daily activities.

**Trial registration:**

ClinicalTrial.gov: NCT00088010

## Background

Back pain is a major public health problem in many countries [1,2]. The high frequency of back pain is pointed out in many studies worldwide. Currently, in the United States, back pain is the leading cause of activity limitation in people younger than 45 years-old, the second most frequent reason for seeking health services, the fifth leading cause of hospitals admission, and the third reason for surgery [3,4]. It is estimated that 70-80% of the world’s population will have at least one episode of back pain in life [5].

Back pain is more common in women (70.3/1000) than in men (68.7/1000) [5], and there is evidence to be a major complaint reported by postmenopausal women [6]. Several factors have been associated with the presence of pain in this period. The decrease of estrogen leads to a bone mass loss, which predisposes to osteoporotic fractures [6]. Sarcopenia also occurs with increasing age, which may cause back pain [7].

High prevalence of vitamin D insufficiency and deficiency in postmenopausal women has been detected in many countries, especially in those with osteoporosis [8]. Low serum 25-hydroxi-vitamin D (25OHD) concentrations reduce the absorption of calcium, leading to decreased bone mass and onset of bone pain [9]. Moreover, the pain may be secondary or a result from the reduction of postural muscles strength, and vitamin D deficiency is a known cause of sarcopenia [10,11]. Vertebral fractures can also cause back pain, once they are more prevalent in individuals with low concentrations of this hormone [12-14]. Another pathophysiologic explanation to associate this disease with back pain is the relationship of inflammation with vitamin D deficiency, once the RANK-RANKL osteoprotegerin system, modulated by vitamin D, has direct link with inflammatory cytokines [15].

### Objective

The primary objective of this paper was to find out if back pain is related to hypovitaminosis D in postmenopausal women with low bone mass.

The secondary objective was to determine if there was difference between postmenopausal women with hypovitaminosis D and with normal serum 25OHD concentrations, regarding the number of bedridden days, number of days away from work and limitation of daily activities.

## Methods

This is a cross-sectional study, based on data previous collected for interventional phase III study in postmenopausal women (CT.gov registration number (NCT00088010) [16]. The randomized, blinded trial, multicenter study was designed primarily to evaluate the effects of a new selective estrogen receptor modulator drug, arzoxifene, on bone fractures and breast cancer. The multicenter study was performed at 232 sites in 23 countries, and complies the same standard of quality. The Eli Lilly and Company, sponsor of the study from which the data were collected, provided data from all research centers and made data available for consultation.

The inclusion criteria for the multicenter study were women aged 60 to 85 years old, at postmenopausal period, with low bone mass. Low bone mass was defined as femoral neck or lumbar spine bone mineral density (BMD), T-score of -1.0 or less. The study excluded women with conditions that influence bone metabolism: unexplained or abnormal vaginal bleeding within 6 months, history of breast cancer or estrogendependent neoplasia, any history of venous thromboembolism, stroke, or transient ischemic attack, liver disease, impaired kidney function, endocrine disorders besides type 2 diabetes, and hypothyroidism that required pharmacologic therapy, bisphosphonates previous treatment, actual systemic corticosteroids use, and clinical vertebral fracture.

For the cross-sectional study, we included all the individuals registered in the multicenter trial. The exclusion criteria for this cross-sectional study were secondary causes of back pain (clinical vertebral fractures, proven neurological problems affecting the spine), diseases that could affect bone metabolism, and use of drugs that interfere with vitamin D metabolism.

Data were collected through a systematic and standardized review. Demographic data, serum 25OHD concentrations, and Newitt-Cummings questionnaires results were collected on screening visit of the multicenter trial.

In the multicenter study, trained professionals collected demographic data and medical history through a structured questionnaire. The blood collection was performed with the individual fasting for 8 hours. The serum 25OHD concentrations were measured using the lab kits NIT5, Covance Central Laboratory, Indianapolis, USA (certified and accredited for medical testing), with the benchmark 25.0 169.7 nmol/L. The Newitt/Cummings questionnaires were answered by the participants with a trained professional in a quiet location. This was a validated questionnaire for back pain [17].

The daily activities were measured according to the Newitt/Cummings questionnaire, which including bending the trunk, lifting a 5 kg object, reaching for an object above the head, putting the socks on, getting in and out of a car, standing about an hour and siting about half an hour.

Postmenopausal women were those whose last menstrual bleeding occurred at least two years ago.

The two populations were determined according to serum 25OHD concentrations, with and without hypovitaminosis D. The Hypovitaminosis D was defined according to Mckenna & Freaney classification [18], which defined vitamin D deficiency as serum 25OHD concentrations below 50 nmol/L.

The Chi-square test (χ2) was used to compare the frequencies between the two populations, once most of the variables were categorical, and odds ratio and coefficient interval (CI) were showed. The Mann-Whitney test was used for numerical variables. The binary logistic regression with “enter” method was used to evaluate if hypovitaminosis D was an independent factor of back pain. For each variable, the entry criterion was p < 0.05, and the exclusion criterion was p > 0.10. The ROC curve was used to determine the best cutoff of 25OHD related to presence of back pain. Statistical analyzes were performed using SPSS version 13.0.

The Ethics Committee of Procardíaco Hospital had already approved the multicenter study, but the Ethics Committee of Fernandes Figueira Institute also approved this study. All the participants signed an informed consent before entering the study.

## Results

The multicenter study included 9354 women. In this cross-sectional study, 49 participants were excluded due to incomplete data; no one was excluded due to clinical vertebral fractures, neurological problems affecting the spine or diseases that could affect bone metabolism and use of drugs that interfere with vitamin D metabolism (once they were already excluded in the multicenter trial). Therefore, 9305 individuals underwent analysis in this study.

The mean age of participants was 67 years (range 60-85 years) and the average age of menopause was 48 years(18-72 years).

Hypovitaminosis D was found in 2272 individuals (24.4%). The vertebral fractures were found in 1412 (15.3%) of the women studied; 67.5% had back pain and 14.8% had limitations in their daily activities in the six previous months.

The Hypovitaminosis D individuals were older, with lower age on menopause and higher body mass index (BMI) than non-hypovitaminosis D women (Table 1).

**Table 1:** Comparison of baseline characteristics between low bone mass postmenopausal women with and without hypovitaminosis D

**Characteristics**	**Hypovitaminosis D**	**Without hypovitaminosis D**	**p-value**
Number of subjects	2272	7033	-
Age (yrs)	67.6 ± 5.8	67.4 ± 5.5	0.035
Age of menopause (yrs)	47.9 ± 5.9	48.2 ± 5.8	0,010
BMI (kg/m^2^)	27.8 ± 4.4	26.7 ± 4.7	0,004

Data displayed as mean ± standard deviation.

*BMI*, Body Mass Index.

Women with hypovitaminosis D reported more back pain than those with regular serum 25OHD concentrations (69.5% vs. 66.9%, p: 0.022), and the pain was more frequent and more severe (Table 2).

**Table 2:** Comparison of back pain and daily activity restrictions between low bone mass postmenopausal women with and without hypovitaminosis D

**Characteristics**	**Hypovitaminosis D**	**Without hypovitaminosis D**	**p-value**	**Odds ratio (CI95%)**
Number of participants	2272	7033	-	-
Back pain in the previous 6 months				
∙Yes	1580 (69.5%)	4704 (66.9%)	0.022	1.14 (1.03 – 1.26)
∙No	682 (30.0%)	2310 (32.8%)		
∙Don’t know	10 (0.4%)	19 (0.3%)		
Pain frequency				
∙All the time	193 (8.5%)	475 (6.8%)	0.004	1.24 (1.03 – 1.48)
∙Almost all the time	330 (14.6%)	1012 (14.4%)		0.96 (0.83 – 1.11)
∙Sometimes	809 (35.7%)	2366 (33.7%)		1.04 (0.92 – 1.16)
∙Rarely	249 (11.0%)	851 (12.1%)		0.85 (0.72 – 0.99)
∙Don’t know	682 (30.1%)	2310 (32.9%)		
Pain severity				
∙Very weak	67 (3.0%)	266 (3.8%)		0.74 (0.56 – 0.98)
∙Weak	363 (16.0%)	1087 (15.5%)	0.001	0.99 (0.86 – 1.14)
∙Moderate	783 (34.6%)	2439 (34.8%)		0.91 (0.81 – 1.02)
∙Severe	322 (14.2%)	807 (11.5%)		1.23 (1.07 – 1.43)
∙Very severe	46 (2.0%)	104 (1.5%)		1.33 (0.92 – 1.91)
∙Don’t know	682 (30.1%)	2310 (32.9%)		
Daily activity restriction in the previous 6 months				
∙Yes	391 (17.2%)	983 (14.0%)	0.001	1.28 (1.12 – 1.46)
∙No	1869 (82.3%)	6006 (85.4%)		
∙Don’t know	12 (0.5%)	42 (0.6%)		

Data displayed as number of participants (%).

*CI*, Confidence Interval.

The Hypovitaminosis D was related to back pain even when analyzed only in individuals without fractures. In this case, 1302 subjects with hypovitaminosis D reported back pain (69.2%), against 3998 women without hypovitaminosis D (66.5%) (p:0.021).

The logistic regression analysis pointed out that presence of hypovitaminosis D was independently related to back pain (p:0.027; Exp(B): 0.890; IC95% 0.802 – 0.987). The ROC curve demonstrated that serum 25OHD concentration below 39.1 had a sensibility of 90% to indicate back pain; concentration above 95.0 had a specify of 90% to rule out back pain. However, the weak area under the curve of 0.525 ± 0.006 (p < 0.001, CI95% 0.512 – 0.538), does not lead to a proper cut off point.

Both populations did not differ regarding back pain localization: cervical pain present in 311 (13.7%) of women with hypovitaminosis D and 974 (13.8%) of those without hypovitaminosis D (p:0.847); thoracic pain was identified in 553 (24.3%) vs. 1594 (22.7%), p:0.099; the thoraco-lumbar transition were referred as painful by 460 (20.2%) vs 1440 (20.5%), p: 0.814; lumbar pain was present in 881 (38.8%) vs 2605 (37.0%), p:0,137. The only region more prevalent in hypovitaminosis D subjects was the lumbar-sacra: 321 (14.1%) vs 876 (12.5%) (p: 0.038).

More hypovitaminosis D subjects reported more daily activities restrictions than those without hypovitaminosis D (17.2% vs 14.0%, p: 0.001). They reported more daily activity restrictions (5.2 +/- 23.3 vs 3.85 +/- 20.4 days, p: <0.001) and longer bedridden (0.7 +/- 5.3 vs 0.3 +/- 3.5 days, p: < 0.001). Moreover, women with hypovitaminosis D had more trouble in performing all daily activities, according to the Newitt/Cummings questionnaire, and the degree of difficulty was higher compared to those with normal serum 25OHD concentrations (Table 3).

**Table 3:** Comparison of daily activity difficulty between low bone mass postmenopausal women with and without hypovitaminosis D

**Daily activity difficulty**	**Hypovitaminosis D**	**Without hypovitaminosis D**	**p-value**	**Odds ratio (CI95%)**
Number of subjects	2272	7033	-	
**Trunk flexion**				
Difficulty				
∙Yes	682 (30.0%)	1676 (23.8%)	<0.001	1.37 (1.24 – 1.53)
∙No	1568 (69.0%)	5298 (75.3%)		
∙Don’t perform this task	22 (1.0%)	58 (0.8%)		
Difficulty level				
∙None	1568 (69.7%)	5298 (76.0%)	<0.001	0.73 (0.65 – 0.81)
∙Some	474 (21.0%)	1209 (17.3%)		1.27 (1.13 – 1.43)
∙Severe	181 (8.0%)	414 (5.9%)		1.39 (1.15 – 1.67)
∙Incapable of	28 (1.2%)	53 (0.8%)		1.64 (1.01 – 2.66)
**Lift a 5 kg object from floor**				
Difficulty				
∙Yes	744 (32.8%)	1846 (26.2%)	<0.001	1.41 (1.27 – 1.57)
∙No	1391 (61.3%)	4880 (69.4%)		
∙Don’t perform this task	136 (6.0%)	307 (4.4%)		
Difficulty level				
∙None	1391 (65.2%)	4881 (72.6%)	<0.001	0.71 (0.64 – 0.79)
∙Some	462 (21.6%)	1234 (18.3%)		1.23 (1.09 – 1.39)
∙Severe	211 (9.9%)	479 (7.1%)		1.43 (1.20 – 1.70)
∙Incapable of	70 (3.3%)	131 (1.9%)		1.71 (1.26 – 2.31)
**Reach for an object above the head**				
Difficulty				
∙Yes	479 (21.1%)	1085 (15.4%)	<0.001	1.49 (1.32 – 1.68)
∙No	1750 (77.0%)	5890 (83.8%)		
∙Don’t perform this task	43 (1.9%)	57 (0.8%)		
Difficulty level				
∙None	1750 (78.5%)	5891 (84.5%)	<0.001	0.67 (0.60 – 0.76)
∙Some	322 (14.5%)	798 (11.4%)		1.31 (1.13 – 1.51)
∙Severe	129 (5.8%)	249 (3.6%)		1.66 (1.33 – 2.08)
∙Incapable of	27 (1.2%)	36 (0.5%)		2.36 (1.39 – 4.01)
**Put own socks on**				
Difficulty				
∙Yes	581 (25.6%)	1513 (21.5%)	<0.001	1.36 (1.22 – 1.52)
∙No	1511 (66.5%)	5353 (76.1%)		
∙Don’t perform this task	179 (7.9%)	166 (2.4%)		
Difficulty level				
∙None	1511 (72.2%)	5353 (78.0%)	<0.001	0.73 (0.66 – 0.82)
∙Some	440 (21.0%)	1178 (17.2%)		1.29 (1.14 – 1.46)
∙Severe	126 (6.0%)	309 (4.5%)		1.36 (1.09 – 1.69)
∙Incapable of	15 (0.7%)	25 (0.4%)		1.98 (0,99 – 3.90)
**Getting in and out of a car**				
Difficulty				
∙Yes	760 (33.5%)	1901 (27.0%)	<0.001	1.38 (1.25 – 1.53)
∙No	1466 (64.6%)	5075 (72.2%)		
∙Don’t perform this task	45 (0.2%)	56 (0.8%)		
Difficulty level				
∙None	1381 (69.6%)	4005 (69.3%)	0.849	1.01 (0.91 – 1.14)
∙Some	526 (26.5%)	1577 (27.3%)		0.96 (0.86 – 1.08)
∙Severe	75 (3.8%)	193 (3.3%)		1.14 (0.86 – 1.50)
∙Incapable of	1 (0.1%)	2 (0.1%)		1.46 (NA)
**Standing up about an hour**				
Difficulty				
∙Yes	1052 (46.3%)	2542 (36.1%)	<0.001	1.56 (1.42 – 1.73)
∙No	1142 (50.3%)	4318 (61.4%)		
∙Don’t perform this task	77 (3.4%)	172 (2.4%)		
Difficulty level				
∙None	1142 (52.1%)	4318 (63.0%)	<0.001	0.64 (0.58 – 0.71)
∙Some	701 (32.0%)	1695 (24.7%)		1.43 (1.29 – 1.59)
∙Severe	298 (13.6%)	724 (10.6%)		1.33 (1.15 – 1.54)
∙Incapable of	52 (2.4%)	122 (1.8%)		1.34 (0.95 – 1.88)
**Sitting down about half an hour**				
Difficulty				
∙Yes	526 (23.2%)	1391 (19.8%)	0.002	1.22 (1.09 – 1.37)
∙No	1737 (76.5%)	5616 (79.9%)		
∙Don’t perform this task	9 (0.4%)	25 (0.4%		
Difficulty level				
∙None	1737 (76.8%)	5616 (80.2%)	0.003	0.82 (0.73 – 0.92)
∙Some	406 (17.9%)	1089 (15.5%)		1.19 (1.05 – 1.35)
∙Severe	116 (5.1%)	283 (4.0%)		1.28 (1.02 – 1.61)
∙Incapable of	4 (0,2%)	18 (0.3%)		0.69 (NA)

Data displayed as number of participants (%).

*NA*, Not Applicable.

*CI*, Confidence Interval.

## Discussion

Back pain is a highly prevalent health problem worldwide. Incidence and prevalence of this symptom are so frequent that it should be studied as an epidemic and social disorder [19]. The body undergoes some changes with aging: projection of the head, shoulders forward, decreased lumbar lordosis, hip, and knee flexion, and increased thoracic curvature (hyperkyphosis). Importantly, the increase in thoracic curvature causes a shift in the center of gravity increasing postural instability and leading to increased susceptibility to falls [20]. Back pain is not perceived as a symptom of menopause, but several factors have been associated with the presence of pain in this period. The decrease of estrogen brings bone mass loss, which predisposes to osteoporotic fractures [6]. Sarcopenia also occurs with increasing age, which may cause back pain [7]. In order to avoid pain, women avoid movements, which in turn lead to muscle fatigability, provoking an undesirable cycle. Ahn S et al studied 9305 postmenopausal women and the prevalence of back pain was 67.5%, the pain was daily in one out of three women [6]. Other studies have similar results. Vogt MT et al also determined a high prevalence of back pain in postmenopausal women; moreover, they established a relationship between this symptom and reduced physical health and more functional limitation [21]. This scenario causes a high absence from work, leading to impact in economy [1,2]. This study showed that almost 15% had daily activities limitations, and 5% were at least one day in bed in the previous 6 months, a number that corroborate to the economic burden caused by this symptom.

This study aimed to evaluate the relationship between vitamin D deficiency with back pain, and therefore the population selected was the most likely to have less concentration of this hormone in the blood. Thus, the study population was low bone mass postmenopausal women with an average of 67 years old and almost one quarter of participants had hypovitaminosis D. This prevalence was demonstrated in previous studies. Lips P et al showed that vitamin D deficiency and insufficiency in postmenopausal women have been detected in many countries and are a growing problem. [8]. Russo LAT et al studied the frequency of inadequate concentrations of vitamin D in low bone mass postmenopausal women in Rio de Janeiro, and found that 27.1% of postmenopausal women had serum 25OHD concentrations below 50 nmol/L [22].

This study compares women with and without hypovitaminosis D and showed a higher frequency of back pain in the first group, statistically significant (70 × 67%, p: 0.022). Despite the small difference of less than 3% in frequency between groups, and therefore not clinically significant, this result points to a relationship between back pain and hypovitaminosis D. Other studies have shown the same association [9,23]. Statistically, a high number of subjects evaluated could lead to non-basis associations. Probably this is not the case, once even excluding subjects with vertebral fractures, the relation between hypovitaminosis D and back pain remains; logistic regression showed that hypovitaminosis D was an independent variable related to back pain. Moreover, the consistency supposed all the data was clear: not only women with hypovitaminosis D had higher frequency of back pain but this pain was significantly more frequent and more severe (pain all the time: 8.5% vs. 6.8%, p:0.004; severe pain or stronger: 16.2% vs 13.0%, p:0.001). Besides this consistency, there is a theoretical basis for the association between the hormone deficiency with back pain. Firstly, sarcopenia, which is characterized by muscle mass and strength loss due to age, is associated with low 25OHD concentrations [10,11]. Another explanation is the modulating impact that vitamin D plays in the RANK-RANKL-osteoprotegerin system, affecting inflammation. This system is linked with pro-inflammatory cytokines, especially tumoral necrose factor α (TNF-α) and interleucine-1 (IL-1), which activate osteoclasts, leading to decreased bone mass [15,24].

The lumbar region was the most commonly affected by pain in the present study. Although it was not expected to find differences in location of pain, postmenopausal women with hypovitaminosis D had more pain in the lumbosacral region compared to those without hypovitaminosis D. This could be explained by the fact that the lumbosacral region is more susceptible to muscle overload [24], and also because sometimes the pain in the lumbar region (which is generally more prevalent) radiates through the buttocks, and therefore the patient reported pain in that location [25].

Another question on the questionnaire addresses daily activities limitation. Aforementioned, studies have established that back pain causes such limitations [21]. The present study showed that there were 3.2% more women with limitations in the group with hypovitaminosis D than the other group. Sarcopenia can justify this finding as it causes muscle strength loss and can be aggravated by age and hypovitaminosis D [10]. Another finding on daily activities limitation is that individuals from the hypovitaminosis D group were longer in bed than the other group: 93.6% did not stay in bed any day against 96.0% (p:0.001); fewer women at hypovitaminosis D group presented with no limitations and the daily activities limitations were longer. A possible explanation is that these limitations were caused by strength decrease due to sarcopenia or by stronger intensity of the inflammation.

Women with hypovitaminosis D had more difficulties in trunk flexion than women with acceptable levels of 25OHD. This movement is probably related to lumbarpelvic imbalance, muscle breakdown between the abdomen, buttocks and lumbar paraspinal muscles, besides the loss of flexibility after 30 years of age [26]. That, coupled with strength loss caused by the sarcopenia, may be affecting these women.

The same happened regarding lifting 5 kg from the ground, reaching for an object above their head, putting on their own socks, getting in and out of a car, standing for about an hour, or siting for about half hour. These movements are based on the same muscle chains, except reaching for an object above their head. Therefore, the groups’ muscles that may have undergone strength reduction are the same, which are paravertebral, multifidus, buttocks, and abdomen. These muscles need to be in balance to play their role as a static and dynamic string. When a muscle does not support the motion, the others try to compensate, causing failure to such balance, and consequently leading to pain [26]. This strength and mass loss were probably caused, once again, by sarcopenia and inflammation.

A Canadian study offered vitamin D supplementation to patients who were referred for surgery due to back pain, and in most of cases they no longer needed to go through surgery [27].

The limitation of this study results from the fact that it is transversal and therefore the concentrations of 25OHD do not necessarily reflect all the six months covered by the survey. Furthermore, by not being prospective, it may have memory bias once the participants had to report back pain in the last six months. This study also did not assess the presence of clinically sarcopenia or inflammation in this population, as they were patients who walked unassisted and therefore unlikely to have these factors detected clinically. Subjects with other obvious cause for back pain were not excluded, once this reason could have its appearance or intensification caused by hypovitaminosis D. Finally, these results could only be related to the population studied, which was postmenopausal women with low bone mass.

## Conclusion

This study pointed out the correlation of hypovitaminosis D with back pain, its frequency, intensity, and activity limitations. Further studies are needed to determine the causal relationship between vitamin D deficiency and back pain, and the role of vitamin D in each of the mechanisms that cause the pain.

## Competing interests

The authors declare that they have no competing interests.

## Authors’ contributions

AVSS – conception, design, acquision of data, analysis and interpretation of data, drafting the manuscript. PGSL – conception, design, acquision of data, analysis and interpretation of data, statics analysis, drafting the manuscript. LATR – conception, design, interpretation of data, final approval of the version to be published. LHG – final approval of the version to be published. RACP – acquision of data, final approval of the version to be published. LPFM - conception, design, interpretation of data, final approval of the version to be published. All authors read and approved the final manuscript.

## Pre-publication history

The pre-publication history for this paper can be accessed here:

http://www.biomedcentral.com/1471-2474/14/184/prepub
